# The impact of carbohydrate quality on gut health: Insights from the NHANES

**DOI:** 10.1371/journal.pone.0315795

**Published:** 2025-02-13

**Authors:** Lanshuo Hu, Xuanchun Huang, Shan Liu, Lihui Fang, Jiaqi Zhang, Xudong Tang

**Affiliations:** 1 Institute of Digestive Diseases, Xiyuan Hospital of China Academy of Chinese Medical Sciences, Beijing, China; 2 Graduate School of Beijing University of Chinese Medicine, Beijing, China; 3 Guang’anmen Hospital, China Academy of Traditional Chinese Medicine, Beijing, China; Jadavpur University, INDIA

## Abstract

**Background:**

High- and low-quality carbohydrate diets are linked to gut health. However, their specific relationship with constipation or diarrhea is unclear. This study uses 2005–2010 NHANES data to examine the relationship between carbohydrate quality and constipation and diarrhea, and to identify suitable populations for different carbohydrate diets.

**Methods:**

Chronic constipation was defined as BSFS types 1 and 2, and chronic diarrhea as types 6 and 7. Dietary intake data were provided by the FPED, using data from the NHANES database. Subjects recalled foods and beverages consumed in the past 24 hours, and intake was averaged and divided into quartiles (Q). After adjusting for covariates, associations between high- and low-quality carbohydrate diets and constipation or diarrhea were assessed using weighted RCS curves and multivariate logistic regression. Results were expressed as weighted ORs and 95% CIs, with subgroup analyses performed.

**Results:**

A total of 11,355 people participated, with 10,488 in the constipation group and 10,516 in the diarrhea group. Multiple regression showed that high-quality carbohydrates were negatively associated with constipation (OR: 0.852, 95% CI: 0.796–0.912, *P* = 0.0001). Low-quality carbohydrates were positively associated with constipation (OR: 1.010, 95% CI: 1.002–1.018, *P* = 0.0295). There was no significant direct association between carbohydrate quality and diarrhoea (*P* = 0.5189, *P* = 0.8278). Segmented regression results showed a non-significant association between low quality carbohydrate intake above 40.65 servings/day and constipation, while quality carbohydrate intake above 3.84 servings/day was not significantly associated with diarrhoea. Subgroup analyses showed differences in carbohydrate quality and constipation or diarrhoea across populations.

**Conclusions:**

High-quality carbohydrates lowered constipation risk by 33.7% and reduced diarrhea risk with intake up to 3.84 servings/day. In contrast, low-quality carbohydrates increased constipation risk by 83.4%, with risk stabilizing beyond 40.65 servings/day. These effects varied across groups, suggesting that better carbohydrate quality supports gut health, especially in sensitive individuals.

## 1. Introduction

Chronic Constipation (CC) and Chronic Diarrhoea (CD) are two of the most common intestinal disorders and are most commonly seen in gastrointestinal outpatient clinics, accounting for approximately one-fifth of the world’s population, with an even higher prevalence in women and the elderly [1–4]. Traditionally, it is believed that CC and CD are primarily associated with poor dietary choices, and therefore dietary therapy is the first line of treatment for CC and CD [5,6].

Carbohydrates are an important part of people’s daily diet, and incorrect consumption of carbohydrates is thought to be an important cause of the development of many diseases in the 21st century. Current evidence suggests that the quality of carbohydrates is a key factor in the ability to achieve healthy outcomes [7,8]. Carbohydrate quality is determined by four key dimensions: dietary fiber intake, whole grain intake, free sugar content, and glycemic index/load. High-quality carbohydrate sources include foods rich in fiber, whole grains, and those low in free sugars and refined starch. These foods—such as whole grains, fruits, and non-starchy vegetables—are digested slowly, resulting in a longer gastrointestinal transit time and stable blood glucose levels [9]. They also provide essential nutrients, including vitamins, minerals, and fiber, which help regulate blood sugar, support cardiovascular health, and meet the body’s nutritional needs. In contrast, low-quality carbohydrates—such as refined grains, potatoes, fruit juices, added sugars, and non-potato root vegetables—are digested quickly and absorbed rapidly, causing spikes in blood glucose [10]. Overconsumption of these foods has been linked to endocrine imbalances, particularly hyperinsulinemia, which can increase appetite, reduce energy expenditure, and elevate the risk of cardiovascular diseases like heart attack, stroke, and even mortality.

Previous carbohydrate quality studies have mostly focused on associations with obesity, diabetes, all-cause mortality and hypertension, while large cross-sectional studies on gut health are lacking [11]. Vitamins and minerals contained in high-quality carbonated water have been found to promote intestinal smooth muscle peristalsis and calcium absorption, with the high fibre also contributing to gut microbial homeostasis, suggesting a potential role of high-quality carbohydrate diets for gut health [12–14]. In contrast, long-term consumption of refined grains and starchy tubers in low-quality carbohydrates may replace dietary fibre, leading to a reduction in intestinal volume and obstruction of stool passage, which can lead to symptoms such as abdominal pain, bloating, constipation, and painful defecation, implying that low-quality carbohydrates may be detrimental to intestinal health [4,15–16]. However, the specific association between carbohydrate quality and the gut is unclear. In addition, the extent to which different quality carbohydrates improve or exacerbate symptoms in different populations with CC or CD is unclear. For example, symptoms of constipation or diarrhoea in diabetic patients may be associated with autonomic neuropathy [17–19], and further research evidence is still needed to determine whether diets with different qualities of carbohydrates can affect the symptoms of CC and CD in this mechanism.

This study introduces a novel approach to understanding the relationship between carbohydrate quality and gut health by utilizing large-scale cross-sectional data from the National Health and Nutrition Examination Survey (NHANES). It addresses limitations in current guidelines, which focus on free sugars and starchy vegetables as low-quality carbohydrates without accounting for the complexity of dietary patterns. By implementing strict dietary controls to reduce confounding factors like fat and energy intake, and conducting subgroup analyses across diverse populations, we overcome the inadequacy of single indicators in measuring carbohydrate quality. Our research questions the assumption that unrestricted intake of high-quality carbohydrates is always beneficial and explores whether a threshold effect exists for gut health improvements. The study’s primary aim is to validate the association between carbohydrate quality and digestive conditions such as constipation and diarrhea, while the secondary goal is to identify specific populations that could benefit from dietary adjustments, emphasizing the clinical relevance of our findings.

## 2. Materials and methods

### 2.1. Study population

The data for this study were sourced from NHANES, operated by the Centers for Disease Control and Prevention (CDC). NHANES is a nationally representative cross-sectional survey conducted by the National Center for Health Statistics (NCHS) using a stratified, multistage probability sampling method to collect health and nutrition data from the U.S. civilian non-institutionalized population. The survey is conducted every two years, and six years (2005–2006, 2007–2008, and 2009–2010) included bowel health information. The Research Ethics Review Board authorized this survey and verified that all participants provided informed consent.

In these three cycles, a total of 31,034 participants were included. However, individuals under 20 years old (n =  13,902), those missing Bowel Health Questionnaire (n =  2,513), and Dietary Questionnaire (n =  1,860) data were excluded. Additionally, 1,404 participants were excluded due to missing other covariates, including marital status (n =  7), PIR (n =  893), education level (n =  6), smoking (n =  2), alcohol consumption (n =  11), BMI (n =  100), PHQ-9 (n =  12), and underlying disease (n =  373).

**Fig 1:**
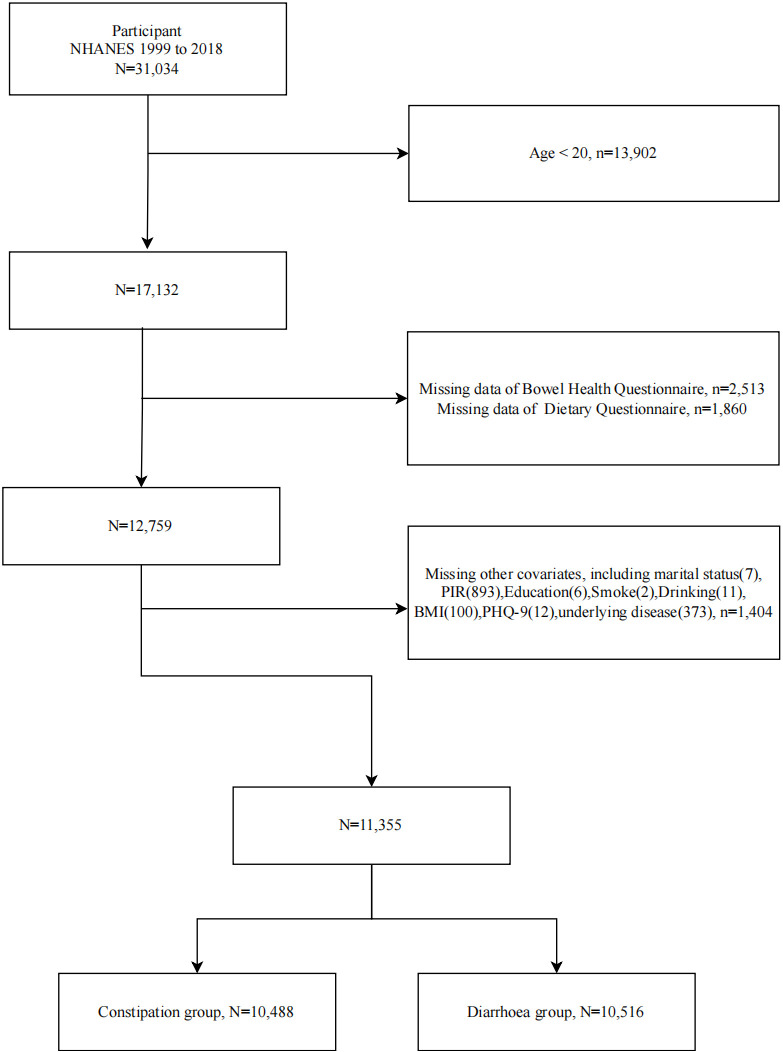
Flowchart of the study population.

Overall, 11,355 participants were included, with 10,488 in the constipation group and 10,516 in the diarrhea group. Detailed statistics can be found at https://www.cdc.gov/nchs/nhanes/. The flowchart is shown in Fig 1. The study protocol was approved by the Research Ethics Review Committee of the National Center for Health Statistics at the Centers for Disease Control and Prevention (CDC), and all NHANES processes were approved by the NCHS Research Ethics Review Board (ERB), besides, participants provided written informed consent. However, as this text involves secondary analysis of data already published in the NHANES database and does not involve patient privacy and safety, it does not require renewed informed consent from participants or ethical approval from the ERB.

### 2.2. Dietary information

All dietary intake data were sourced from the Food Patterns Equivalents Database (FPED). The FPED provides nutritional values of foods, including quantities of fruits, vegetables, grains, protein foods, dairy, oils, added sugars, solid fats, and alcoholic beverages. The first dietary recall interview was conducted at a Mobile Examination Center (MEC), and the second interview was conducted via telephone 3–10 days later. Participants were asked to recall all food and beverages consumed in the past 24 hours (from midnight to midnight). The average food intake over these two days was calculated.

To better explore the relationship between dietary carbohydrates and symptoms of constipation and diarrhea, daily carbohydrate intake was divided into high-quality and low-quality carbohydrate intake. The daily intake of high-quality and low-quality carbohydrates was calculated by summing specific food groups from the Food Patterns Equivalents Database (FPED). High-quality carbohydrate intake includes whole grains, legumes, whole fruits, and non-starchy vegetables, while low-quality carbohydrate intake includes refined grains, fruit juices, starchy vegetables, and added sugars. Each carbohydrate type is measured in FPED using specific units based on its category: whole grains and refined grains are measured in “ounce equivalents”; legumes, whole fruits, juices, non-starchy vegetables, and starchy vegetables are measured in “cup equivalents”; and added sugars are measured in “teaspoon equivalents.” Detailed intake amounts are provided in S1 Table.

### 2.3. Bowel health questionnaire

Participants’ chronic diarrhea or constipation status was determined based on their responses to the Bowel Health Questionnaire. Researchers presented participants with a card featuring color images and descriptions of the Bristol Stool Form Scale (BSFS, types 1–7) for reference. Consistent with previous studies, participants whose usual or most common stool type was classified as BSFS type 1 (separate hard lumps, like nuts) or BSFS type 2 (sausage-like but lumpy) were categorized as having chronic constipation. Those whose usual or most common stool type was classified as BSFS type 6 (fluffy pieces with ragged edges, a mushy stool) or BSFS type 7 (watery, no solid pieces) were categorized as having chronic diarrhea. The remaining participants were classified as having normal bowel habits.

### 2.4. Covariates

We adjusted for risk factors associated with chronic diarrhea and chronic constipation [20,21]. Covariates included in this study were age, sex, race/ethnicity (non-Hispanic white, non-Hispanic black, and other races), education level (≤high school, >high school), marital status (living alone, married or living with a partner), poverty income ratio (PIR; range 0–5, measuring socioeconomic status, categorized as PIR < 2 or ≥2), smoking status (never smoked or smoked <  100 cigarettes in their lifetime, smoked ≥ 100 cigarettes and currently smoking), alcohol consumption (defined as drinking if a person consumes at least 12 drinks per year), and vigorous physical activity (in the 2005–2006 cycle, defined as engaging in at least 10 minutes of vigorous activity in the past 30 days that caused heavy sweating or a significant increase in breathing or heart rate. From 2007–2010, those who answered “yes” to the question “Does your work involve vigorous physical activity that causes a significant increase in breathing or heart rate, such as carrying or lifting heavy loads, digging, or construction work for at least 10 minutes?” were considered to engage in vigorous physical activity). Body mass index (BMI) was calculated as weight in kilograms divided by height in meters squared, classified as underweight/normal (<25 kg/m²), overweight (25–30 kg/m²), and obese (≥30 kg/m²). Diabetes status was defined by a doctor’s diagnosis, HbA1c > 6.5%, fasting glucose >  7.0 mmol/l, random glucose ≥11.1 mmol/l, 2-hour OGTT glucose ≥11.1 mmol/l, or current medication use. Hypertension was defined by current use of antihypertensive medication, a doctor’s diagnosis, systolic blood pressure over 130 mmHg, or diastolic blood pressure over 80 mmHg. Other variables included coronary heart disease, hyperlipidemia, cancer, and depression (Patient Health Questionnaire-9 (PHQ-9) score ≥10, a validated measure for depression), and dietary intake assessed by trained interviewers for total fiber, fat, water, and energy intake.

### 2.5. Statistical analysis

We used R (version 4.4.0, 2024, R Foundation; http://www.r-project.org). Using NHANES census data from 2005 to 2010, we examined baseline characteristics of study individuals through descriptive analysis, stratified by the presence of constipation and diarrhea. Continuous variables were presented as weighted means ±  standard errors (SE), while categorical variables were presented as counts (weighted percentages, %). Categorical variables were analyzed using weighted chi-square statistics, and continuous variables were analyzed using analysis of variance (ANOVA). We calculated six-year weights based on the Bowel Health Questionnaire. Multivariable logistic regression models estimated the odds ratios (ORs) and 95% confidence intervals (CIs) between high or low-quality carbohydrate intake and chronic diarrhea and constipation. We constructed three models: the crude model (unadjusted), Model 1 adjusted for age, sex, race, marital status, PIR, and educational level, and Model 2 further adjusted for smoking, drinking, BMI, and physical activity. Model 3 included additional adjustments for diabetes, hypertension, CHD, hyperlipidemia, cancer, depression, dietary fiber intake (g/day), total fat intake (g/day), and total water intake (g/day). We used restricted cubic splines (RCS) with spline smoothing functions to assess dose-response relationships between high or low-quality carbohydrate intake and the risk of chronic diarrhea and constipation. Finally, we conducted subgroup analyses and interaction tests to explore potential differences across various populations. All significance tests were two-sided, with *p* <  0.05 considered statistically significant.

## 3. Results

### 3.1. Basic characteristics of the study population

When analyzing constipation as the outcome, 10,488 participants were included after excluding various factors. Among them, 839 had constipation, while 9,649 were healthy. The average age of those with constipation was 46.071 years, and the average high-quality carbohydrate intake was 2.766 ±  0.050 servings per day.

When analyzing diarrhea as the outcome, 10,516 participants were included, with 867 having diarrhea and 9,649 being healthy. The average age of those with diarrhea was 50.597 ±  0.748 years. Their average high-quality carbohydrate intake was 2.595 ±  0.124 servings per day, and the average low-quality carbohydrate intake was 23.887 ±  0.755 servings per day.

The overall prevalence of constipation was 8.0%, and the overall prevalence of diarrhea was 8.2%. In the constipation group, compared to healthy individuals, those with constipation were more likely to be non-Hispanic Black, female, have lower income, lower education levels, or be depressed. They also had lower energy intake, lower water consumption, did not engage in vigorous physical activity, had lower fiber intake, and tended to consume alcohol.

The univariate analysis of the diarrhea group showed that, compared to healthy individuals, those with diarrhea were more likely to be female, older, have lower income, lower education levels, not engage in vigorous physical activity, be obese, consume alcohol, smoke, be depressed, have diabetes, hypertension, hyperlipidemia, or cancer. Table 1 summarizes the characteristics of the participants.

**Table 1:** Based on the baseline characteristics of the study population ascertained.

	Constipation				Diarrhea			
	Total	Normal	constipation	*P*	Total	Normal	diarrhea	*P*
**n**	10488	9649	839		10516	9649	867	
**N (weighted)**	142107096	131774008	10333088		141787233	131774008	10013224	
**Age, year**	47.062 ± 0.357	47.140 ± 0.379	46.071 ± 0.597	0.124	47.384 ± 0.382	47.140 ± 0.379	50.597 ± 0.748	<0.0001
**PIR**	3.135 ± 0.042	3.161 ± 0.041	2.804 ± 0.081	<0.0001	3.142 ± 0.041	3.161 ± 0.041	2.888 ± 0.080	<0.001
**BMI, kg/m** ^**2**^	28.648 ± 0.122	28.722 ± 0.125	27.699 ± 0.301	0.001	28.844 ± 0.119	28.722 ± 0.125	30.448 ± 0.303	<0.0001
**Energy intake, kcal/day**	2127.577 ± 13.966	2146.324 ± 14.594	1888.506 ± 24.586	<0.0001	2140.946 ± 14.493	2146.324 ± 14.594	2070.173 ± 43.067	0.082
**Dietary fiber, g/day**	16.558 ± 0.193	16.746 ± 0.192	14.168 ± 0.344	<0.0001	16.686 ± 0.188	16.746 ± 0.192	15.898 ± 0.517	0.117
**Total fat, g/day**	80.823 ± 0.699	81.716 ± 0.737	69.435 ± 1.025	<0.0001	81.553 ± 0.731	81.716 ± 0.737	79.410 ± 2.097	0.273
**Total water, g/day**	1958.137 ± 37.067	1985.993 ± 38.241	1602.891 ± 65.536	<0.0001	1982.304 ± 38.213	1985.993 ± 38.241	1933.748 ± 94.936	0.567
**High-quality carbohydrates, serving/day**	2.766 ± 0.050	2.809 ± 0.050	2.210 ± 0.075	<0.0001	2.794 ± 0.050	2.809 ± 0.050	2.595 ± 0.124	0.087
**Low-quality carbohydrates, serving/day**	24.421 ± 0.283	24.436 ± 0.278	24.225 ± 0.555	0.641	24.397 ± 0.280	24.436 ± 0.278	23.887 ± 0.755	0.445
**Sex**				<0.0001				<0.001
Male	5245 (48.740)	4993 (50.443)	252 (27.022)		5366 (49.886)	4993 (50.443)	373 (42.562)	
Female	5243 (51.260)	4656 (49.557)	587 (72.978)		5150 (50.114)	4656 (49.557)	494 (57.438)	
**Race**				<0.0001				0.068
Non-Hispanic White	5518 (73.777)	5132 (74.299)	386 (67.119)		5546 (74.037)	5132 (74.299)	414 (70.583)	
Non-Hispanic Black	2043 (10.227)	1840 (9.862)	203 (14.884)		2018 (9.983)	1840 (9.862)	178 (11.582)	
Other Race	2927 (15.996)	2677 (15.839)	250 (17.998)		2952 (15.980)	2677 (15.839)	275 (17.834)	
**Marital Status**				0.002				0.671
Non-single	6511 (66.312)	6039 (66.797)	472 (60.125)		6571 (66.737)	6039 (66.797)	532 (65.953)	
Single	3977 (33.688)	3610 (33.203)	367 (39.875)		3945 (33.263)	3610 (33.203)	335 (34.047)	
**Education**				<0.0001				<0.0001
<=high school	5151 (40.284)	4659 (39.572)	492 (49.368)		5186 (40.366)	4659 (39.572)	527 (50.815)	
>high school	5337 (59.716)	4990 (60.428)	347 (50.632)		5330 (59.634)	4990 (60.428)	340 (49.185)	
**Smoke**	2223 (21.018)	2058 (21.070)	165 (20.350)	0.003	2267 (21.490)	2058 (21.070)	209 (27.016)	<0.001
**Drinking**	7084 (73.266)	6585 (73.864)	499 (65.639)	<0.001	7112 (73.387)	6585 (73.864)	527 (67.117)	0.004
**Vigorous Physical activity**	2489 (26.649)	2329 (27.111)	160 (20.755)	0.004	2490 (26.659)	2329 (27.111)	161 (20.709)	0.003
**Diabetes**	1795 (12.426)	1652 (12.485)	143 (11.675)	0.521	1862 (12.934)	1652 (12.485)	210 (18.846)	<0.0001
**Hypertension**	5538 (48.373)	5131 (48.643)	407 (44.942)	0.093	5669 (49.054)	5131 (48.643)	538 (54.464)	0.032
**Cardiovascular disease**	448 (3.340)	414 (3.388)	34 (2.730)	0.26	454 (3.387)	414 (3.388)	40 (3.373)	0.984
**Hyperlipidemia**	7514 (70.814)	6929 (70.880)	585 (69.971)	0.631	7615 (71.285)	6929 (70.880)	686 (76.619)	0.002
**Cancer**	1025 (9.173)	937 (9.035)	88 (11.100)	0.145	1057 (9.343)	937 (9.035)	120 (13.566)	<0.001
**Depression**	807 (6.408)	703 (6.064)	104 (10.793)	<0.0001	851 (6.711)	703 (6.064)	148 (15.227)	<0.0001

### 3.2. Relationship between high or low quality carbohydrates and gut health

#### 3.2.1. Relationship between high/low quality carbohydrates and constipation.

Table 1 shows a association between high-quality carbohydrate intake and constipation. To explore this relationship further, a multivariable logistic regression analysis was conducted. After multiple adjustments, it was found that a diet high in quality carbohydrates was negatively associated with constipation (OR: 0.852, 95% CI: 0.796, 0.912, *P* =  0.0001).

Moreover, when using high-quality carbohydrate intake as a stratified variable (quartiles), further analysis in the fully adjusted model indicated that individuals in the highest quartile (Q4) had a lower risk of constipation compared to those in the lowest quartile (Q1) (OR: 0.663, 95% CI: 0.508, 0.864, *P* =  0.0061, *P* for trend =  0.0006). This reinforces the beneficial link between high-quality carbohydrates and reduced constipation risk.

Conversely, low-quality carbohydrate intake was significantly positively associated with the risk of constipation. After similar adjustments, low-quality carbohydrates were found to have a positive association with constipation risk (OR: 1.010, 95% CI: 1.002, 1.018, *P* =  0.0295). Furthermore, individuals in the highest quartile (Q4) of low-quality carbohydrate intake had a significantly higher risk of constipation compared to those in the lowest quartile (Q1) (OR: 1.834, 95% CI: 1.381, 2.435, *P* =  0.0004, *P* for trend =  0.0008).

Trend tests revealed that with each additional serving of high-quality carbohydrates, there was a statistically significant decrease in constipation risk. Conversely, increased intake of low-quality carbohydrates was associated with a statistically significant increase in constipation risk (see Table 2 for details).

**Table 2:** Adjusted OR [95% confidence interval (CI)] for the association between constipation and carbohydrate mass quartiles.

	Quartiles	Model 1^a^	*P*	Model 2^b^	*P*	Model 3^c^	*P*	Model 4^d^	*P*
		OR (95%CI)		OR (95%CI)		OR (95%CI)		OR (95%CI)	
**High-quality carbohydrates**		0.849 (0.805, 0.896)	<0.0001	0.869 (0.823, 0.918)	<0.0001	0.853 (0.809, 0.899)	<0.0001	0.852 (0.796, 0.912)	0.0001
**High-quality carbohydrates**	Q1	Ref.		Ref.		Ref.		Ref.	
	Q2	0.778 (0.606, 1.000)	0.0561	0.794 (0.617, 1.022)	0.0819	0.775 (0.602, 0.998)	0.0575	0.829 (0.650, 1.056)	0.1437
	Q3	0.560 (0.454, 0.691)	<0.0001	0.597 (0.480, 0.743)	<0.0001	0.561 (0.453, 0.696)	<0.0001	0.635 (0.512, 0.787)	0.0004
	Q4	0.539 (0.427, 0.681)	<0.0001	0.599 (0.469, 0.767)	0.0002	0.552 (0.434, 0.701)	<0.0001	0.663 (0.508, 0.864)	0.0061
***P* for trend**		<0.0001		<0.0001		<0.0001		0.0006	
**Low-quality carbohydrates**		1.026 (1.021, 1.032)	<0.0001	1.019 (1.013, 1.026)	<0.0001	1.020 (1.013, 1.027)	<0.0001	1.010 (1.002, 1.018)	0.0295
**Low-quality carbohydrates**	Q1	Ref.		Ref.		Ref.		Ref.	
	Q2	1.354 (1.077, 1.701)	0.0128	1.256 (1.004, 1.572)	0.0538	1.225 (0.979, 1.532)	0.0859	1.153 (0.918, 1.447)	0.2334
	Q3	1.918 (1.410, 2.608)	0.0002	1.699 (1.264, 2.284)	0.0012	1.664 (1.233, 2.246)	0.0022	1.453 (1.059, 1.994)	0.0303
	Q4	3.048 (2.429, 3.826)	<0.0001	2.426 (1.926, 3.057)	<0.0001	2.449 (1.929, 3.110)	<0.0001	1.834 (1.381, 2.435)	0.0004
***P* for trend**		<0.0001		<0.0001		<0.0001		0.0008	

^a^Adjust Energy intake.

^b^Adjust Model 1 + Age, Sex, Race, marital status, PIR, and educational level.

^c^Adjust Model 2 + Smoke, Drinking, BMI, Physical activity.

^d^Adjust Model 3 + Diabetes, Hypertension, CHD, hyperlipidemia, Cancer, Depression, Dietary fiber intake, Total Fat intake, Total Water.

#### 3.2.2. Relationship between high/low quality carbohydrates and diarrhoea.

After adjusting for the weighted data in the crude model, this study found no direct association between high or low-quality carbohydrate diets and diarrhea (High-quality carbohydrates: 95% CI: 0.890, 1.060, *P* =  0.5189; Low-quality carbohydrates: 0.999, 95% CI: 0.990, 1.008, *P* =  0.8278). However, trend tests showed a statistically significant decrease in diarrhea with each additional serving of high-quality carbohydrates (*P* for trend =  0.0100). In contrast, increased intake of low-quality carbohydrates did not show a significant trend with diarrhea (*P* for trend =  0.9644) (see Table 3 for details).

**Table 3:** Adjusted OR [95% confidence interval (CI)] for the association between diarrhoea and carbohydrate mass quartiles.

	Quartiles	Model 1^a^	*P*	Model 2^b^	*P*	Model 3^c^	*P*	Model 4^d^	*P*
		OR (95%CI)		OR (95%CI)		OR (95%CI)		OR (95%CI)	
**High-quality carbohydrates**		0.953 (0.890, 1.020)	0.1709	0.950 (0.878, 1.027)	0.2068	0.965 (0.893, 1.043)	0.3797	0.971 (0.890, 1.060)	0.5189
**High-quality carbohydrates**	Q1	Ref.		Ref.		Ref.		Ref.	
	Q2	0.820 (0.640, 1.050)	0.1236	0.791 (0.608, 1.030)	0.0915	0.811 (0.627, 1.048)	0.119	0.796 (0.605, 1.049)	0.1188
	Q3	0.673 (0.495, 0.917)	0.0157	0.633 (0.459, 0.874)	0.0089	0.667 (0.484, 0.918)	0.0189	0.640 (0.457, 0.897)	0.0163
	Q4	0.665 (0.497, 0.889)	0.0086	0.624 (0.449, 0.866)	0.0081	0.676 (0.496, 0.920)	0.0187	0.607 (0.404, 0.912)	0.0248
***P* for trend**		0.0032		0.0031		0.0097		0.01	
**Low-quality carbohydrates**		1.003 (0.995, 1.012)	0.4349	1.002 (0.992, 1.011)	0.7509	1.001 (0.992, 1.011)	0.7877	0.999 (0.990, 1.008)	0.8278
**Low-quality carbohydrates**	Q1	Ref.		Ref.		Ref.		Ref.	
	Q2	1.109 (0.876, 1.405)	0.3959	1.100 (0.863, 1.402)	0.448	1.124 (0.881, 1.434)	0.354	1.111 (0.878, 1.407)	0.3907
	Q3	1.104 (0.834, 1.462)	0.4928	1.098 (0.828, 1.457)	0.5212	1.124 (0.836, 1.511)	0.4433	1.070 (0.804, 1.424)	0.6453
	Q4	1.109 (0.784, 1.569)	0.5625	1.071 (0.746, 1.537)	0.7125	1.073 (0.745, 1.545)	0.7082	0.980 (0.694, 1.384)	0.9092
***P* for trend**		0.5763		0.6971		0.6834		0.9644	

^a^Adjust Energy intake.

^b^Adjust Model 1 + Age, Sex, Race, marital status, PIR, and educational level.

^c^Adjust Model 2  + Smoke, Drinking, BMI, Physical activity.

^d^Adjust Model 3 + Diabetes, Hypertension, CHD, hyperlipidemia, Cancer, Depression, Dietary fiber intake, Total Fat intake, Total Water.

### 3.3. Restrictive cubic spline curves between high or low quality carbohydrates and constipation/diarrhoea

In Table 3, dietary carbohydrate quality is significantly associated with constipation, though there might be a non-linear relationship between the quality of dietary carbohydrates and the severity of constipation. To further explore the specific relationship between high or low-quality carbohydrates and constipation, we used weighted restricted cubic splines (RCS) to assess the associations between high/low-quality carbohydrates and constipation and diarrhea. The variables in Model II in Table 3 were also adjusted.

**Fig 2:**
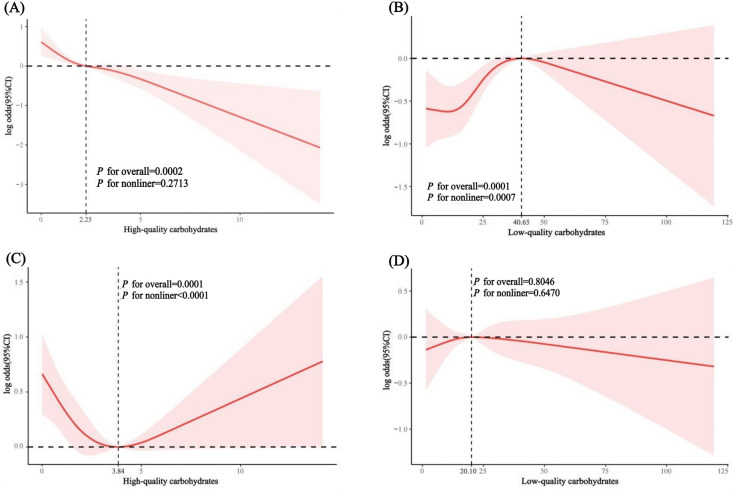
Restricted Cubic Spline Analysis of High- and Low-Quality Carbohydrate Intake and Symptoms of Constipation and Diarrhea. (A) Relationship between high-quality carbohydrate intake and constipation. (B) Relationship between low-quality carbohydrate intake and constipation. (C) Relationship between high-quality carbohydrate intake and diarrhea. (D) Relationship between low-quality carbohydrate intake and diarrhea.

The RCS results (Fig 2) indicate a negative association between a high-quality carbohydrate diet and constipation, but no significant non-linear relationship (overall P-value =  0.0002, non-linear P-value =  0.2713). Conversely, low-quality carbohydrates showed a positive association with constipation symptoms, with a significant non-linear relationship (overall P-value <  0.0001, non-linear P-value =  0.0007). The relationship between low-quality carbohydrates and constipation was found to be an inverted U-shape, with segmented regression revealing a significant positive association when the intake of low-quality carbohydrates was less than 40.65 servings/day (OR: 1.022, 95% CI: 1.011, 1.033, *P* =  0.0008), but it flattened out beyond 40.65 servings/day.

A high-quality carbohydrate diet was negatively correlated with diarrhea and exhibited a significant non-linear relationship (overall *P*-value =  0.0001, non-linear *P*-value <  0.0001), whereas the intake of low-quality carbohydrates did not show a significant relationship with diarrhea (overall *P*-value =  0.8046). Since the RCS curve for high-quality carbohydrates was U-shaped, further segmented regression found that the significant negative association was present up to a daily intake of 3.84 servings (OR: 0.853, 95% CI: 0.741, 0.981, *P* =  0.0360), but it flattened out beyond 3.84 servings/day (see Tables 4 and 5 for details).

**Table 4:** Threshold Effects Between Low-Quality Carbohydrates and Constipation.

	LOW categorical ≤ 40.65	LOW categorical ≥ 40.65	*P*-interaction
**Low-quality carbohydrates**	1.022 (1.011, 1.033)	0.986 (0.970, 1.002)	0.0001

**Table 5:** Threshold Effects Between High-Quality Carbohydrates and Diarrhea.

	HIGH categorical ≤ 3.84	HIGH categorical > 3.84	*P*-interaction
**High-quality carbohydrates**	0.853 (0.741, 0.981)	1.099 (0.994, 1.215)	0.0022

The horizontal axis represents dietary intake, and the vertical axis represents the relationship (β coefficient) between carbohydrate quality and symptoms of constipation and diarrhea.

### 3.4. Subgroup analysis of high/low quality carbohydrates and constipation

**Fig 3:**
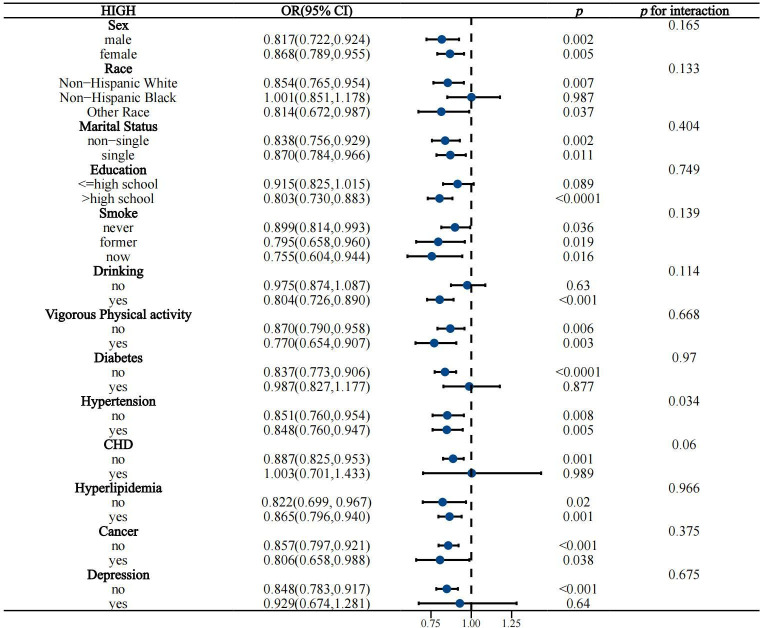
Subgroup analysis of categorical variables related to high-quality carbohydrates and constipation.

**Fig 4:**
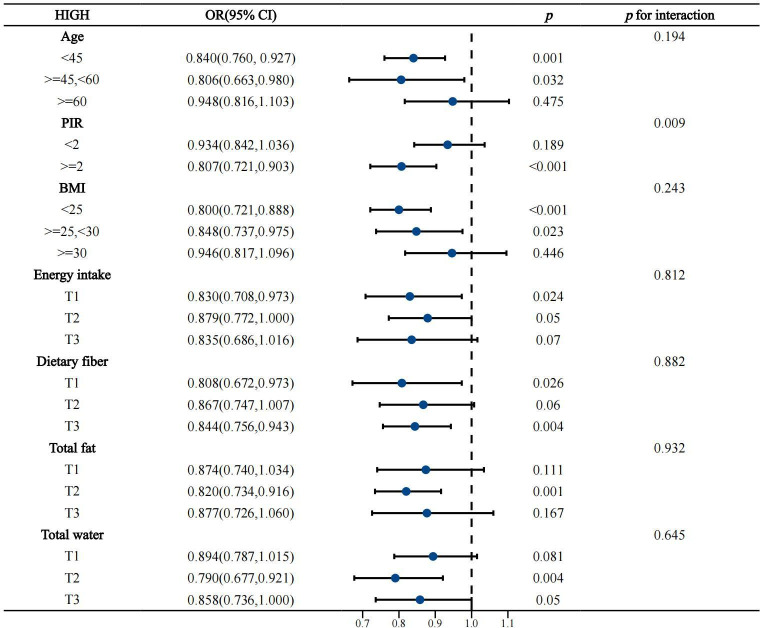
Subgroup analysis of continuous variables related to high-quality carbohydrates and constipation.

**Fig 5:**
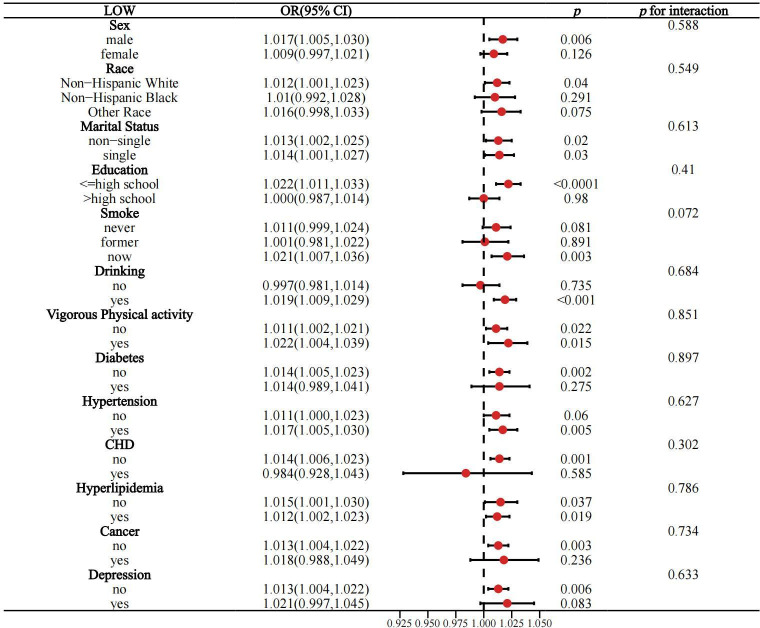
Subgroup analysis of categorical variables related to low-quality carbohydrates and constipation.

**Fig 6:**
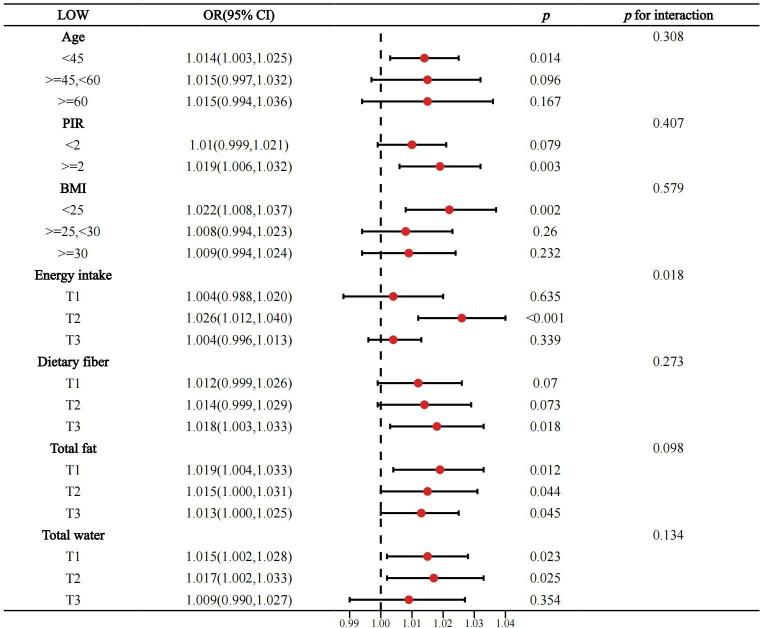
Subgroup analysis of continuous variables related to low-quality carbohydrates and constipation.

The above results show a significant association between high/low-quality carbohydrates and constipation. To further understand whether the effects of high or low-quality carbohydrates on constipation vary significantly among different subgroups (such as age, gender, BMI, etc.), and to better control and adjust for potential confounding factors to ensure the accuracy and reliability of the study results, a subgroup analysis was conducted (see Figs 3–6).

The subgroup analysis showed that the negative association between high-quality carbohydrates and constipation was consistent across various subgroups categorized by gender, race, age, BMI, education level, smoking, drinking, diabetes, vigorous activity, coronary heart disease, hyperlipidemia, cancer, depression, energy intake, total water intake, total fat intake, and total fiber intake. However, this negative association was not significant in subgroups with low education levels, non-drinkers, individuals with diabetes, coronary heart disease, depression, those older than 60 years, PIR <  2, BMI >  30, medium to high energy intake, medium fiber intake, and those with very low or very high fat and water intake.

The positive association between low-quality carbohydrates and constipation was generally consistent across all subgroups, but was less pronounced in subgroups of women, non-Hispanic Blacks and other races, those with high education levels, never or former smokers, non-drinkers, individuals with diabetes, non-hypertensive individuals, those with coronary heart disease, cancer, depression, those older than 45 years, PIR <  2, BMI >  25, those with very high or very low energy intake, low to medium fiber intake, and those with excessive water intake. Overall, the model was robust and minimally affected by specific populations.

### 3.5. Sensitivity analyses

To address the potential impact of other dietary components on gut health, we conducted a sensitivity analysis on the relationship between carbohydrate quality and the incidence of constipation and diarrhea. Specifically, we controlled for factors such as fat, protein, and energy intake in addition to the original covariates. The results indicated that the statistical effect of carbohydrate quality on diarrhea and constipation remained highly significant. For further details, please refer to Table 6.

**Table 6:** Sensitivity analysis of the impact of carbohydrate quality on gut health.

	Diarrhea OR (95%CI) *P*-value	Constipation OR (95%CI) *P*-value
**High-quality carbohydrates**	0.975 (0.891, 1.067) 0.5920	0.851 (0.795, 0.911) 0.0001
**High-quality carbohydrates quartile**		
Q1	Ref.	Ref.
Q2	0.803 (0.608, 1.061) 0.1376	0.828 (0.649, 1.056) 0.1437
Q3	0.646 (0.460, 0.907) 0.0199	0.633 (0.512, 0.782) 0.0004
Q4	0.620 (0.409, 0.939) 0.0348	0.660 (0.502, 0.868) 0.0072
***P* for trend**	0.0142	0.848 (0.781, 0.920) 0.0006
**Low-quality carbohydrates**	1.001 (0.991, 1.010) 0.9146	1.011 (1.002, 1.021) 0.0261
**Low-quality carbohydrates**		
Q1	Ref.	Ref.
Q2	1.127 (0.887, 1.432) 0.3402	1.166 (0.930, 1.461) 0.1974
Q3	1.093 (0.820, 1.457) 0.5522	1.483 (1.086, 2.025) 0.0218
Q4	1.016 (0.718, 1.436) 0.9300	1.913 (1.430, 2.560) 0.0003
***P* for trend**	0.8742	1.240 (1.118, 1.377) 0.0005

Adjust: Age, Sex, Race, marital status, PIR, and educational level, Smoke, Drinking, Energy intake BMI, Physical activity, Diabetes, Hypertension, CHD, hyperlipidemia, Cancer, Depression, Dietary fiber intake, Total Fat intake, Total Water, Total Protein.

## 4. Discussion

This study was based on the NHANES large cross-sectional data and included a total of 11,355 participants whose intake was assessed for its association with bowel health by carbohydrate quality score. The results of multivariate logistic regression showed that high-quality carbohydrate intake was negatively associated with constipation; while low-quality carbohydrate intake was positively associated with constipation and was significant at low-quality carbohydrate intake of less than 40.65 servings/day. High-quality carbohydrate intake was negatively associated with diarrhoea and was significant until high-quality carbohydrate intake was less than 3.84 servings/day. It is evident that high quality carbohydrates (whole grains and fibre) have a bi-directional modulatory effect on gut health [22,23] and the effect of different quality carbohydrates within a given intake on gut health is significant [24].

The effects of high or low quality carbohydrate diets on the gut may be achieved through several mechanisms: (1) Influence on fiber intake: High-quality carbohydrates are rich in dietary fiber, which are fermented in the colon by the microbiota to produce gas and short-chain fatty acids (SCFAs) [25], such as butyrate. Butyrate is not only an important source of energy for the colonic mucosa, but also increases intestinal peristalsis [26–28]. Low-quality carbohydrates exacerbate constipation because they are usually deficient in dietary fibre, and insufficient fibre leads to reduced intestinal peristalsis and dry stools that are difficult to pass. At the same time, loss of dietary fibre, which reduces the production of SCFAs, increases the permeability of the colonic mucus barrier, triggering inflammation and pathogen invasion [29,30]. (2) Altering water content: faecal consistency is closely related to water content, and small changes in water content can significantly affect faecal movement and emptying [31]. High-quality carbohydrates (vegetables and fruits) contain more water, and fibre also retains water and increases faecal hydration, thereby promoting defecation [32]. In addition, excess fibre absorbs large amounts of water in the gut, further exacerbating the symptoms of diarrhoea, which may explain the limited value of the positive effect of high quality carbohydrates on diarrhoea. (3) Anti-inflammatory effects: high-quality carbohydrate diets have been associated with lower levels of inflammation. High-fibre diets reduce inflammation by altering intestinal pH and permeability [33,34]. There is a association between dietary inflammatory potential and the composition of the gut microbiota, and higher grain and fibre intake may reduce chronic inflammation, whereas low-quality carbohydrates, such as over-refined sugars, increase oxidative responses and promote the persistence of chronic inflammation [35]. (4) Adjusting amino acid and vitamin intake: high-quality carbohydrates are rich in folate and other vitamins, nutrients that are essential for neurological health, and amino acids such as tryptophan help regulate mood and well-being [36]. Deficiencies in these nutrients may increase the risk of depression, affect gut health in the context of the gut-brain axis, and develop functional intestinal disorders [37]. (5) Alteration of gut microbial communities: the type of carbohydrate can shape different gut microbial communities. High-quality carbohydrates support beneficial flora by providing fibre, whereas low-quality carbohydrates (high in free sugars) disturb the flora balance and shift the metabolism of the gastrointestinal microbiota towards the production of harmful metabolites, leading to constipation and diarrhoea [38,39].

In this study, we further understood the effects of high or low quality carbohydrates on constipation in different subgroups of the population, and subgroup analyses revealed that the association between different quality carbohydrates and constipation was not significant in PIR < 2, middle-aged and elderly populations, non-drinking alcohol, obesity, diabetes mellitus, coronary artery disease, depression, and excessive water intake populations.

The reasons for this may be the lack of nutritional knowledge in low-income populations, the inability to access sufficient high-quality food resources, and the poor understanding and implementation of a healthy diet, which affects the effectiveness of high-quality carbohydrate intake. Additionally, there may be a lack of resources or capacity for constipation management and treatment due to poor medical care, resulting in the problem of constipation relying more on improvements in lifestyle habits rather than just adjustments in dietary composition [40]. Furthermore, low-income populations might be more likely to consume low-quality carbohydrates because they often opt for cheaper, calorie-dense foods to satisfy hunger. These foods, such as fried chicken, fries, and potatoes, are typically more affordable but nutritionally inferior compared to high-quality food options. This dietary pattern can contribute to a higher intake of low-quality carbohydrates and exacerbate issues like constipation.. Compared to younger people, middle-aged and older adults may have more underlying diseases, weakened digestive system, reduced intestinal motility, and possibly a slower response to carbohydrates [41]. Drinking behaviour may lead to deranged energy metabolism, and carbohydrates are an important energy supplement for drinkers and have a marked effect on them. However, non-drinkers may have a relatively balanced energy intake and a healthier digestive system, which in turn diminishes the effect of carbohydrate mass on constipation [42,43]. People who are overweight or obese may influence gut health through a number of factors, including high-fat and high-protein diets, and the accompanying metabolic disorders and chronic inflammatory states in obese patients may influence the role of carbohydrate mass on gut health [44]. Gut motility in diabetic patients may be affected by autonomic neuropathy, and when autonomic neuropathy leads to slowing of gut motility, on the one hand, it is prone to small intestinal bacterial overgrowth, which leads to bile salt catabolism, fat malabsorption, and thus diarrhoea; on the other hand, the slowing of gut motility may weaken or eliminate gastrocolic reflexes after eating, which slows down the rate of colonic transit, leading to constipation, and thus weakening the the potential benefits of high-quality carbohydrates on gut health, while the role for the effects of low-quality carbohydrates thus becomes more ambiguous [45,46]. Patients with coronary artery disease are often accompanied by multiple chronic conditions, and medication and the condition itself may have a complex impact on bowel function, such as the use of diuretics and calcium channel blockers, which often interfere with normal faecal excretion, mitigating the effects of carbohydrates [47]. Depression exists with altered dietary habits and may choose foods that are high in sugar, fat and low in fibre, which often bring euphoria but tend to lead to constipation. Depression is often associated with low physical activity, which also slows down bowel motility, while psychological factors influence the imbalance of the intestinal flora and affect the function of the digestive system. Side effects of certain antidepressants such as sertraline and citalopram include constipation [48,49].

In addition, the association between low-quality carbohydrates and constipation was not significant in female, race, or cancer populations. Women may be more concerned with healthy eating, even when consuming low-quality carbohydrates, and they may alleviate constipation through other dietary modifications [50,51]. There are differences in gut microbiota and dietary habits across races, and geographic/racial differences in microbiome structure attributed to differences in host genetics and innate/adaptive immunity influence the role of low-quality carbohydrates on constipation [52]. While cancer patients’ diets are carefully modified and they may take more anticancer drugs to meet specific nutritional and therapeutic needs and mitigate the effect of low-quality carbohydrates on constipation, high-quality carbohydrates are significantly negatively correlated with constipation in cancer populations, probably because high-quality carbohydrates help metabolise toxins from the faeces and reduce their negative effects [53]. In terms of energy intake, this study found that too high or too low energy intake may lead to adjustments in other dietary structures (e.g., protein and fat intake) to counteract the negative effects of low-quality carbohydrates [54]. People with moderate fibre intake did not achieve optimal fibre intake, affecting the ameliorative effect of high-quality carbohydrates on constipation. Inadequate fibre intake may contribute to constipation, but the effects of low quality carbohydrates may be masked by other dietary factors [55].

The FODMAP diet, which stands for fermentable oligosaccharides, disaccharides, monosaccharides, and polyols, includes carbohydrates that are difficult to absorb in the small intestine and ferment in the large intestine, producing gas and attracting water, leading to bloating, abdominal pain, and diarrhea [56]. The FODMAP diet is well-supported by evidence for treating Irritable Bowel Syndrome (IBS). IBS patients often experience constipation and diarrhea. Reducing the intake of easily fermentable carbohydrates can lower intestinal gas and osmolality, improving gastrointestinal function. These fermentable polysaccharides are often seen as low-quality carbohydrates, negatively affecting intestinal health. In contrast, low FODMAP foods rich in fiber and high-quality carbohydrates can significantly alleviate IBS-C symptoms, aligning with this study’s recommendations. The quality of carbohydrates also relates to individual tolerance to different FODMAPs, with varying tolerance levels among individuals [57]. This aligns with the subgroup analyses in this paper, where dietary treatments are both generalized and specific. For patients with multiple dietary restrictions, such as vegetarians or diabetics, creating a balanced and nutritious diet is clinically important [58].

In conclusion, our study is the first to elucidate the significant relationship between carbohydrate quality and digestive issues such as constipation and diarrhea. We found that high-quality carbohydrates—rich in dietary fiber and whole grains, and low in free sugars and glycemic index—are more crucial for gut health than merely reducing carbohydrate intake. This challenges the conventional endorsement of low-carb or ketogenic diets, which often lead to gut imbalances. Our findings advocate for a dietary shift towards high-quality carbohydrates to prevent gut issues and support weight management, offering a balanced alternative to restrictive diets. Additionally, high-quality carbohydrates can alleviate both constipation and diarrhea, benefiting individuals with mixed-type IBS. These insights have profound clinical implications, particularly in enteral nutrition therapy, where soluble fibers and SCFAs are essential for preventing and treating diarrhea. This research paves the way for more effective nutritional strategies to enhance gut health, leading to improved decision-making in clinical settings and a reduction in societal health burdens. Our study emphasizes the importance of carbohydrate quality in both clinical and everyday dietary practices.

However, there are some limitations to our study. Firstly, there are many potential factors affecting diet, the systemic physiological effects of high and low quality carbohydrates are not yet fully understood, and although we included covariates such as hypertension, diabetes mellitus, hyperlipidaemia, and cancer, we were unable to exclude the effects of all potential confounders, and therefore the findings need to be validated in subsequent studies. Second, cross-sectional studies, although useful in initial exploration and hypotheses, cannot provide causal evidence and lack external validation, which may lead to biased results. Future studies should adopt more longitudinal designs or experimental methods for a deeper understanding of the problem. Finally, there are significant differences in genetic backgrounds, lifestyles, healthcare levels, and socioeconomic conditions across countries and regions, and the findings of this study may not be fully applicable to other countries. Therefore, caution is needed when applying these results to other regions of the globe, taking into account unique environmental and demographic characteristics. Future studies should expand the sample to cover more countries and cultures to enhance the generalisability and applicability of the results.

## 5. Conclusions

High-quality carbohydrates positively impact gut health by reducing risks of constipation and diarrhea. In the top quartile of high-quality carbohydrate intake, constipation risk was 33.7% lower, showing a linear negative trend. Low-quality carbohydrates, however, increased constipation risk by 83.4% in the highest intake group, with a U-shaped trend where risk rose below 40.65 servings/day but stabilized beyond. For diarrhea, high-quality carbohydrates showed a protective effect up to 3.84 servings/day, while low-quality carbohydrates had no significant association. Enhancing carbohydrate quality appears to be an effective strategy to support gut health, especially for sensitive populations.

## Supporting information

S1 Table Per reference unit of high-quality carbohydrates and low-quality carbohydrates. (XLSX)

S2 Table Primary data on the relationship between constipation and carbon and water quality. (CSV)

S3 Table Primary data on the relationship between diarrhea and carbon and water quality. (CSV)

## Abbreviation

CC: Chronic Constipation
CD: Chronic Diarrhoea
FPED: Food Patterns Equivalents Database
BMI: Body Mass Index
PIR: poverty income ratio
RCS: restricted cubic splines
PHQ-9: Patient Health Questionnaire-9
SCFAs: short-chain fatty acids.
